# Engineering random spin models with atoms in a high-finesse cavity

**DOI:** 10.1038/s41567-023-02033-3

**Published:** 2023-05-04

**Authors:** Nick Sauerwein, Francesca Orsi, Philipp Uhrich, Soumik Bandyopadhyay, Francesco Mattiotti, Tigrane Cantat-Moltrecht, Guido Pupillo, Philipp Hauke, Jean-Philippe Brantut

**Affiliations:** 1 Institute of Physics and Center for Quantum Science and Engineering, Ecole Polytechnique Fédérale de Lausanne (EPFL), Lausanne, Switzerland; 2 Pitaevskii BEC Center, CNR-INO and Dipartimento di Fisica, Università di Trento, Trento, Italy; 3 INFN-TIFPA, Trento Institute for Fundamental Physics and Applications, Trento, Italy; 4 University of Strasbourg and CNRS, CESQ and ISIS (UMR 7006), aQCess, Strasbourg, France

**Keywords:** Quantum simulation, Phase transitions and critical phenomena

## Abstract

All-to-all interacting, disordered quantum many-body models have a wide range of applications across disciplines, from spin glasses in condensed-matter physics over holographic duality in high-energy physics to annealing algorithms in quantum computing. Typically, these models are abstractions that do not find unambiguous physical realizations in nature. Here we realize an all-to-all interacting, disordered spin system by subjecting an atomic cloud in a cavity to a controllable light shift. Adjusting the detuning between atom resonance and cavity mode, we can tune between disordered versions of a central-mode model and a Lipkin–Meshkov–Glick model. By spectroscopically probing the low-energy excitations of the system, we explore the competition of interactions with disorder across a broad parameter range. We show how disorder in the central-mode model breaks the strong collective coupling, making the dark-state manifold cross over to a random distribution of weakly mixed light–matter, ‘grey’, states. In the Lipkin–Meshkov–Glick model, the ferromagnetic finite-sized ground state evolves towards a paramagnet as disorder is increased. In that regime, semi-localized eigenstates emerge, as we observe by extracting bounds on the participation ratio. These results present substantial steps towards freely programmable cavity-mediated interactions for the design of arbitrary spin Hamiltonians.

## Abstract (web summary)

Random spin models play a key role in our understanding of disorder and complex many-body systems. Two all-to-all interacting, disordered models have now been realized using a cavity quantum electrodynamics platform.

## Main

The unavoidable presence of impurities and inhomogeneities in most real-world physical systems has given a strong motivation to the study of disordered models. In such studies, important insights into the typical behaviour of a many-body system can be obtained by considering an ensemble of realizations with randomly distributed parameters^1^. In this way, a deeper understanding of the structure of low-energy excitations in complex quantum systems can be achieved, providing keys to interpreting transport and thermodynamics observations. Going one step further, several quantum simulation platforms, such as trapped ions^2^, ultracold atoms^3^ and Rydberg atoms^4–6^, have demonstrated the capability to implement controlled disorder into otherwise clean many-body systems. Those allowed for the investigation of non-equilibrium dynamics, revealing some of the most intriguing phenomena of random systems, such as Anderson^7–11^ and many-body localization^12–14^.

In the last years, cavity quantum electrodynamics (QED) has emerged as a new platform for quantum simulation. By harnessing photons to tailor novel types of interaction beyond the native van der Waals and dipolar interactions between atoms, cavity QED unites the scalability of atom devices with tunable long-range interactions^15^. Previous experiments used this platform to explore new, superradiant^16–19^ and dissipation-stabilized^20,21^ phases of matter in quantum gases, as well as to demonstrate tunable-range interactions^22^ and emergent geometries using spatial and spectral addressing^23^.

In this article, we implement random spin models on a cavity QED platform and study their low-lying excitations. Via a light-shift technique, we realize a quasi-random longitudinal field with controlled strength, which competes with an all-to-all flip–flop interaction mediated by the exchange of cavity photons. Leveraging the open nature of the cavity, we observe the frequency-resolved response in the cavity field and atomic polarization channels. We exploit our setup to observe disorder-driven crossovers in two different regimes: a central-mode model where we observe a disorder-induced dressing of otherwise dark antisymmetric states with cavity photons, and a Lipkin–Meshkov–Glick (LMG) model (an instance of a Richardson–Gaudin model) where disorder competes with ferromagnetic order. As shown theoretically and experimentally, the frequency-resolved susceptibilities are sensitive to the detailed structure of excitations, providing particular insights into their localization properties. Our system is a natural starting point to investigate the spectacular consequences of strong light–matter coupling on materials properties^24–26^ such as transport^27–29^ or magnetism^30^, where the effect of disorder due to impurities and material inhomogeneities is believed to be strongly influenced by light.

## Model

Our system implements a paradigmatic model consisting of *N* Ising spins, mapped to internal atomic states, identically coupled to the central, bosonic photon mode of the cavity. By exposing the *i*th spin to a random energy shift *ϵ*_*i*_, the model is described by the disordered Tavis–Cummings-type Hamiltonian as

1 $${\hat{H}}_{{{{\rm{TC}}}}}={{{\varDelta }}}_{{{{\rm{ca}}}}}{\hat{a}}^{{\dagger} }\hat{a}+g\sqrt{N}\left({\hat{S}}^{+}\hat{a}+{\hat{S}}^{-}{\hat{a}}^{{\dagger} }\right)+\mathop{\sum }\limits_{i=1}^{N}{\epsilon }_{i}\,\frac{{\hat{\sigma }}_{i}^{z}}{2}.$$

Here ${\hat{a}}^{{\dagger} }$ and $\hat{a}$ are the creation and annihilation operators of the photons in the cavity, ${\hat{\sigma }}_{i}^{r}$ are the *r*-Pauli operators acting on the Ising (pseudo-)spin-1/2 of the *i*th atom, ${\hat{S}}^{+(-)}=\mathop{\sum }\nolimits_{i = 1}^{N}{\hat{\sigma }}_{i}^{+(-)}/\sqrt{N}$ are the collective spin-raising (lowering) operators and ${{{\varDelta }}}_{{{{\rm{ca}}}}}$ is the detuning between the cavity and bare atomic resonance. We set *ℏ* = 1 throughout the manuscript. Central-mode models^31,32^ have been used to describe a large variety of physical situations, including qubit decoherence in solid-state quantum computing platforms as well as heat and charge transport in nanostructures.

**Fig. 1:**
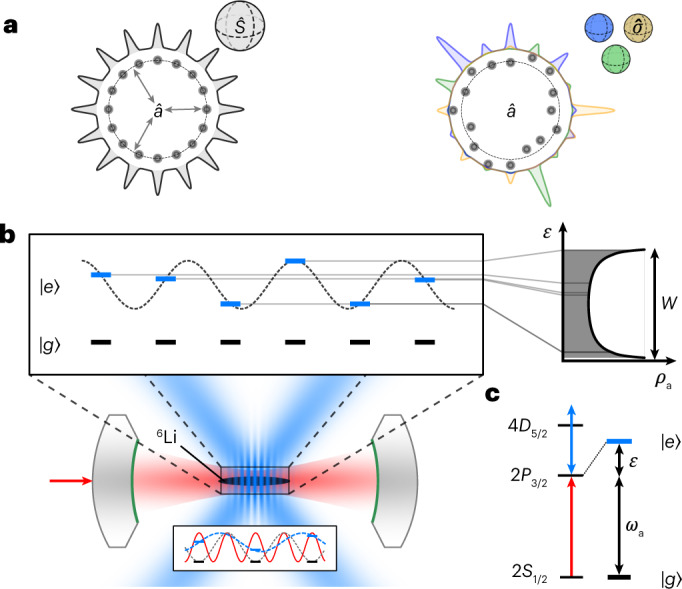
Concept of the experiment. **a**, Fragmentation of collective light–matter eigenstates with increasing disorder. Disorder-free system with all the spins (spheres) identically coupled to the central mode $\hat{a}$ provided by the cavity field, forming a symmetric collective Dicke state (left). With disorder, the collective state fragments into few- or single-spin ensembles whose constituents are located at arbitrarily large distances, exchanging excitations through the cavity, sketched here for three excitation modes (right). **b** Experimental realization: atoms are trapped in an optical resonator, forming an atom array commensurate with the cavity mode, ensuring identical atom–light coupling. Two crossed light-shifting beams (blue) illuminate the atoms with an incommensurate standing-wave inference pattern, leading to a quasi-random intensity distribution *ρ*_a_ over the atoms (right). The inset below illustrates the positions of the atoms (black bars) with respect to the cavity-field intensity (red wave), the optical dipole trapping potential (grey wave) and the intensity of the light-shifting lattice (blue wave). **c**, Simplified level diagram of the ^6^Li atoms. The light-shifting laser (blue arrow) off-resonantly couples the 2*P*_3/2_ manifold with the higher-lying 4*D*_5/2_ manifold, yielding a dressed state |*e*〉 (blue), with an energy shift proportional to the laser intensity.

In the disorder-free instance of the Hamiltonian of equation (1) (Fig. 1a, left), the spin-1/2 degrees of freedom form a manifold of *N* + 1 collective exchange-symmetric Dicke states coupled to light, thus called ‘bright states’, which are described by a single collective spin $\hat{S}$. The remaining 2^*N*^ − (*N* + 1) states form a dark manifold that is decoupled from the cavity field. In the single-excitation manifold, this structure reduces to two polaritons and *N* − 1 dark states. A controlled breaking of this collective spin description into macroscopic subsets that are spatially and spectrally distinguishable has recently been demonstrated by splitting atomic ensembles with the help of optical tweezers and magnetic-field gradients^23^.

In the model of equation (1), the collective spin description is broken by disorder (Fig. 1a, right). This leads to the fragmentation of the dark-state manifold into an ensemble of ‘grey eigenstates’ that are hybridizations of the delocalized photon field as well as a few localized spins with similar energies^27^. Because the coupling to the cavity extends over the entire system, energy resonances between spins can occur at arbitrarily large distances in the presence of disorder. As a result, the grey eigenstates have wavefunctions that are neither localized nor delocalized but semi-localized over multiple, arbitrarily distant spins^33,34^. It was recently demonstrated theoretically that for any strength of light–matter coupling, this results in a multifractal structure of the eigenstates, similar to that found at the critical points of localization–delocalization transitions^35^. Even though they have never been directly observed, it is believed that disorder-induced grey states are responsible for the spectacular enhancement in energy and charge transport found in disordered molecular systems coupled to cavities^27,28,36–40^.

**Extended Data Fig. 1:**
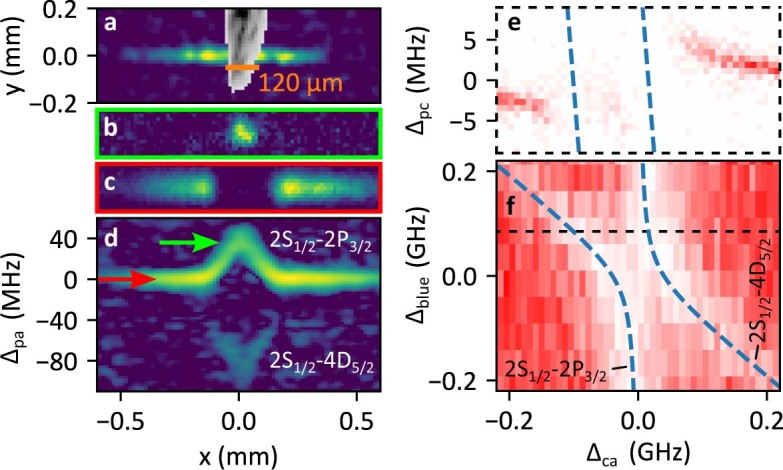
Characterization of light-shift of excited state. **a**, Absorption picture of an atomic cloud in the cavity dipole trap. The grey needle in the middle is used to shield central atoms from the absorption light, making it possible to empty the outer pancake traps using the radiation pressure force of the absorption imaging. **b** and **c**, Absorption image of the cloud at maximal shift (green arrow in **d**) and zero shift (red arrow in **d**). **d**, Spatially-resolved absorption spectroscopy of the D2 transition in the presence of a single lattice beam (Gaussian beam centred on *x* = 0 mm with waist of 120μm). Each horizontal line of plot **d** is the *y*-integrated optical density. The light shifting laser was 50 MHz blue detuned from the 2*P*_3/2_–4*D*_5/2_ transition. We can observe both resonances of the Autler–Townes doublet, the strong light-shifted single photon transition (top) and the faint two-photon transition (bottom). **e**, Cavity transmission spectroscopy for cloud illuminated with homogenous light-shifting beam. The dashed blue lines indicate the expected resonance frequencies of the Autler–Townes doublet. **f**, 2D map of cavity transmission spectra for different detunings of the light-shifting laser from the 2*P*_3/2_–4*D*_5/2_ transition. Each horizontal line is a cavity transmission spectrum. In the colorbar, red indicates a large number of photons transmitted through the cavity. The black horizontal dashed line marks the configuration of panel **e**.

Experimentally, the Hamiltonian in equation (1) is realized by an array of *N* = 90–800 thermal ^6^Li atoms confined in about 160 trapping sites, positioned at the anti-nodes of the resonant cavity field. The spins are encoded in the $2{S}_{1/2}^{F = 1/2}$ (|*g*〉) and 2*P*_3/2_ (|*e*〉) states of ^6^Li atoms (Fig. 1b,c). The cavity resonance is tuned close to the 2*S*_1/2_–2*P*_3/2_ transition at 671 nm, with the detuning given by ${{{\varDelta }}}_{{{{\rm{ca}}}}}$. Our cavity is close to concentric, leading to a single-atom cooperativity of *η* = (4*g*^2^)/(*κΓ*) = 6.4, where *g*/2π, *κ*/2π and *Γ*/2π are 2.05, 0.45 and 5.80 MHz. Due to the cloud’s temperature of 200 μK, and the reduced dipole moment for linearly polarized light at zero magnetic field, the average cavity coupling that the atoms experience is $\bar{g}/2\uppi$ = 1.23 MHz (Methods and Extended Data Fig. 1a–c,e).

The disorder is created by two laser beams that intersect at the position of the atoms, with frequency slightly detuned from the 2*P*_3/2_–4*D*_5/2_ transition at 460 nm (Extended Data Fig. 1d,f), forming a light-shifting lattice with a period of 1.04 μm that is incommensurate with the trapping lattice, which has a period of 671 nm. This produces a quasi-random pattern of strong light shifts in the 2*P*_3/2_ state, with negligible effect on atoms in the ground state (Fig. 1b,c). These light shifts result in quasi-disordered energy shifts *ϵ*_*i*_, which translate into the spin language as random local longitudinal fields sampled from the distribution ${\rho }_{{{{\rm{a}}}}}(\epsilon )={[\uppi \sqrt{\epsilon (W-\epsilon )}]}^{-1}$, where *W* is proportional to the intensity of the control laser (Methods). We neglect the light shifts induced by the trapping light on the ground and excited states, as it is small compared with the light shift induced by the 460 nm beam.

**Fig. 2:**
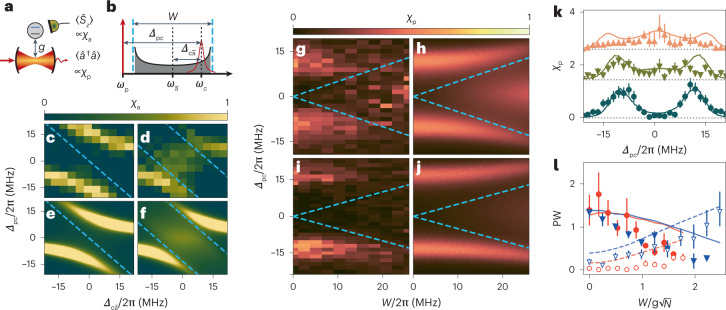
Response of the system in the central-mode regime. **a**, Measurement of atomic and photonic susceptibilities on a drive of the cavity. Photonic susceptibility *χ*_p_ is given by a cavity transmission measurement, whereas atomic susceptibility *χ*_a_ is proportional to the number of atoms that have been excited by the cavity field (Methods). **b**, Frequency diagram illustrating the relative detunings between the atoms with average frequency ${\omega }_{\overline{{{{\rm{a}}}}}}$ in range *W*, the cavity at frequency *ω*_c_ and probe at *ω*_p_. The light-blue dashed lines indicate the edges of the atomic frequency distribution. In all the other panels, the atomic states lie between the two light-blue lines. **c**–**f**, Measured (**c** and **d**) and simulated (**e** and **f**) (see the ‘Susceptibility in the near-resonant regime’ section, where *N* = 100 atoms) atomic susceptibility maps as a function of atom–cavity and pump–cavity detunings (*x* and *y* axis, respectively), for the clean system (**c** and **e**) and at maximal disorder *W*/2π = 26 MHz (**d** and **f**). **g**–**j**, Measured (**g** and **i**) and simulated (**h** and **j**) photonic susceptibility as a function of disorder strength *W* for different atom numbers: *N* = 74 (**g** and **h**) and *N* = 145 (**i** and **j**). **k**, Vertical sections of **g** and **h** overlapped (curves are vertically offset for clarity) for $W/g\sqrt{N}=0,1,2$. **l**, Photon weight (PW) of the grey states (empty markers, dashed lines) and polaritons (filled markers, continuous lines) as a function of normalized disorder strength for *N* = 145 (circles) and *N* = 74 (triangles) atoms, indicating the disappearance of polaritons and appearance of grey states. The grey states’ photon weight was measured by taking the average photonic susceptibility over the grey-state region defined by ${{{\varDelta }}}_{{{{\rm{pc}}}}}$ ∈ { −*Γ*/2, *Γ*/2}, whereas the photon weight of the polariton was quantified by taking the height of the lower polariton in **g** and **i**, which is not affected by the radiation pressure of the light-shifting beam (Methods). In **k**, data are presented as mean values ± standard error of the mean (s.e.m.). Averages run over 106 measurements. In **l**, data are presented as mean values ± s.e.m. Averages run over 106 measurements for *N* = 145 (circles) and 113 measurements for *N* = 74 (triangles).

**Extended Data Fig. 2:**
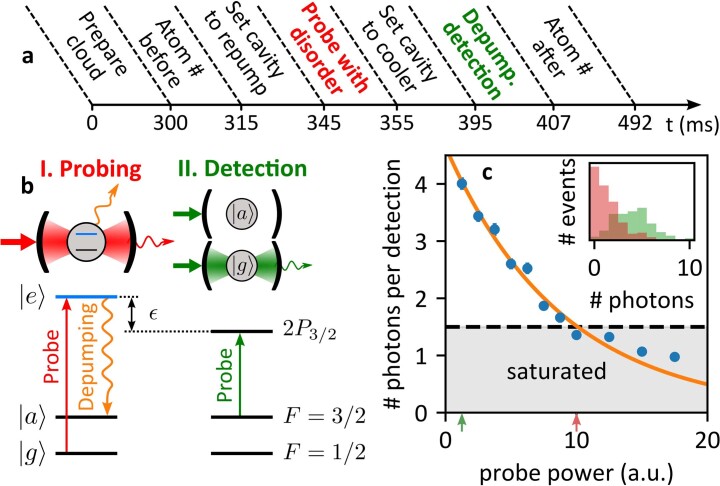
Experimental sequence and measurement of atomic susceptibility. **2c**: data are presented as mean values +/- SEM, Averages run over 371 measurements. **a**, Timeline of the experimental sequence. The core elements are the interrogation of the disordered cavity–atom system (red) and the subsequent detection of depumped atoms in the $\left\vert a\right\rangle$(*F* = 3/2) state. **b**, Illustration of the probe configurations during interrogation and depumping detection. **I**, When probing the disordered system, the excited state of the atoms is dressed with the light-shifting laser, indicated by the blue colour of the level and the shift *ϵ*. The photons entering the cavity from the probe have two decay channels. Either they leak out of the cavity on the other side (red wiggled arrow) where they will be detected by a single photon counter, or they can be lost by free-space spontaneous emission of an atomic excitation (orange wiggled line). At zero-magnetic field, the transition from $\left\vert g\right\rangle$ to $\left\vert e\right\rangle$ is not closed, therefore spontaneous emission events can depump the atom into the $\left\vert a\right\rangle$ state. **II**, These depumped atoms can be detected by measuring the cavity transmission. If the cavity is on resonance with the $\left\vert a\right\rangle$–$\left\vert e\right\rangle$ transition and only a single atoms is in state $\left\vert a\right\rangle$ the transmission gets strongly suppressed. **c**, Calibration of depumping signal. The mean number of transmitted photons during the depumping detection is plotted against the probe power during the interrogation of the disordered system. The error bars of the data represent statistical fluctuations. The orange line shows a fit of the theoretically expected relation [see Eq. (8)]. The inset shows a histogram of detected photons for low (green) and high (red) probe powers (see arrows on *x*-axis for configuration).

We probe the system by weakly driving the cavity on axis with a probe beam and measuring both photon transmission proportional to $\langle {\hat{a}}^{{\dagger} }\hat{a}\rangle$ and atomic excitations $\langle {\hat{S}}^{z}\rangle =\langle \mathop{\sum}\nolimits_{i = 1}^{N}{\hat{\sigma}}_{i}^{z}\rangle/(2N)$ using an optical pumping technique (Fig. 2a). In the linear-response regime, this provides us with the frequency-dependent photonic and atomic (spin) susceptibilities, namely, *χ*_p_ and *χ*_a_, respectively (equations (5), (7) and (15) provide the definitions and Extended Data Fig. 2 provides the details).

## Near-resonant regime and grey states

We first investigate the regime at small ${{{\varDelta }}}_{{{{\rm{c}}}}\bar{{{{\rm{a}}}}}}$ where the cavity resonance is close to the mean atomic resonances, that is, ${{{\varDelta }}}_{{{{\rm{c}}}}\bar{{{{\rm{a}}}}}}={{{\varDelta }}}_{{{{\rm{ca}}}}}-W/2$ (Fig. 2b). In the absence of disorder, we observe the canonical normal-mode splitting of a width $2g\sqrt{N}/2\pi$ of 22 MHz expected from the Tavis–Cummings model (Fig. 2c). As a result of this splitting, a Rabi gap forms at ${{{\varDelta }}}_{{{{\rm{c}}}}\bar{{{{\rm{a}}}}}}=0$, and direct atomic excitations at the bare resonance frequency are suppressed (Fig. 2c, centre). Although there are *N* − 1 eigenstates of the Hamiltonian lying within the gap, these are purely atomic, and the symmetry of the all-to-all atom–cavity coupling prevents their excitation, rendering them completely dark.

On introducing disorder, we observe the onset of a non-zero response around zero detuning, a manifestation of the increase in photon weight of the originally dark purely atomic states. A representative spectrum of *χ*_a_ for *W*/(2π) = 26 MHz is presented in Fig. 2d. We observe that the fading out of Rabi splitting occurs via a redistribution of the spectral weight from the polaritons to a wide spectrum of midgap states. For $| {{{\varDelta }}}_{{{{\rm{c}}}}\bar{{{{\rm{a}}}}}}| \gtrsim W$, a narrow, dispersively shifted cavity resonance is restored at around ${{{\varDelta }}}_{{{{\rm{pc}}}}}$ = 0 (Fig. 2d).

To further understand the evolution of the spectrum with disorder strength, we probe the photonic susceptibility at ${{{\varDelta }}}_{{{{\rm{c}}}}\bar{{{{\rm{a}}}}}}=0\,$ as a function of disorder strength *W* and detuning ${{{\varDelta }}}_{{{{\rm{pc}}}}}$. The results are presented in Fig. 2g,i for different mean atom numbers *N*. For weak disorder, photonic susceptibility *χ*_p_ confirms the presence of two bright polaritons, and a manifold of degenerate dark states at the centre of the Rabi gap. As the disorder becomes comparable with the collective atom–cavity coupling, that is, $W \approx g\sqrt{N}$, we observe a smooth increase in *χ*_p_ at around ${{{\varDelta }}}_{{{{\rm{pc}}}}}$ = 0, signalling the onset of a finite coupling of a grey-state manifold emerging from the originally dark states. Simultaneously, the polaritons’ response weakens and fades away for the largest disorder, where the spectrum consists of a resonance centred at ${{{\varDelta }}}_{{{{\rm{pc}}}}}$ = 0 strongly broadened by the disorder.

The evolution of the spectrum with disorder is driven by the fragmentation of the eigenstates, from fully delocalized bright and dark states without disorder, to randomly distributed, isolated resonances for the largest disorder. To confirm this interpretation, we compare our observation (Fig. 2g,i) with theoretical calculations (Fig. 2h,j) of the cavity transmission based on Green function techniques (see the ‘Susceptibility in the near-resonant regime’ section). The model takes into account the experimental distribution of spin energies, which is correlated and non-uniform, different from the case studied elsewhere^35^.

Nevertheless, we have verified that the eigenfunctions are multifractal in the same way (Supplementary Section 1.1). The simulations (which take into account the measured atom number fluctuation), the effects of thermal motion on atom–cavity couplings and both losses of photons and atomic decay, are in good agreement with the observations for the low-disorder regime. For the strongest disorder, deviations particularly appear for the upper polariton, whose signal appears moderately weaker in the experiment. We attribute this to losses induced by radiation pressure from the control laser at 460 nm, predominantly affecting the excited atoms with the largest admixture in the 4*D*_5/2_ manifold (Methods). For the largest disorder strength, we do not resolve the polaritons themselves but observe a clear signal from the grey states. These results are further confirmed in Fig. 2k, which presents a direct comparison of the experimental and theoretical data for photonic susceptibility *χ*_p_ as a function of ${{{\varDelta }}}_{{{{\rm{pc}}}}}$ for representative values of disorder strength *W*. The same simulation procedure also reproduces atomic susceptibility *χ*_a_ measured as a function of detunings (Fig. 2e,f).

We quantitatively analyse the fading out of the polariton and the emergence of grey states by comparing the photonic susceptibility in the lower (respectively middle) parts of the spectrum (Fig. 2g–j). This yields the overall photon weight of the polariton and grey states as a function of normalized disorder strength (Fig. 2l). The crossover between the light–matter-interaction-dominated regime and disorder-dominated regime is evident as spectral weight is smoothly transferred from the polariton to grey states, in qualitative agreement with the simulations.

## Large-detuning regime and LMG model

**Fig. 3:**
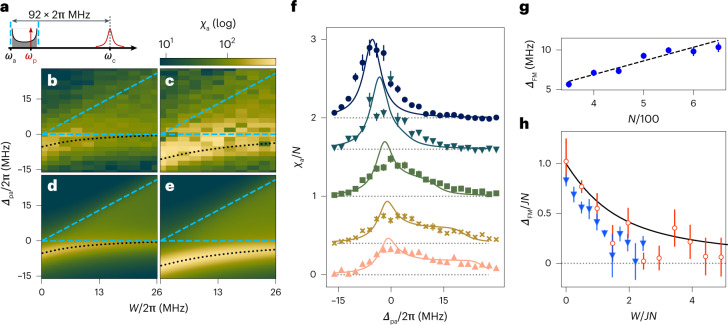
Response of the random LMG model. **a**, Frequency diagram depicting the detuning among atomic disorder, cavity and weak cavity probe. **b**–**e**, Measured (**b** and **c**) and simulated (**d** and **e**) atomic susceptibility for *N* = 303 (**b** and **d**), and *N* = 610 (**c** and **e**) atoms. **f**, Cuts through **b** and **d**, illustrating the quantitative agreement between the experiment (markers) and theory (solid lines). The cuts show data for different disorder strengths *W*/2π = 0.0, 5.2, 13.0, 20.8 and 26.0 MHz (top to bottom), and are offset from one another according to (26 − *W*/2π)/13. **g**, Scaling of the collective ferromagnetic gap ${{{\varDelta }}}_{{{{\rm{FM}}}}}$ = *JN* at zero disorder *W* = 0 with mean atom number *N*. **h**, Behaviour of ${{{\varDelta }}}_{{{{\rm{FM}}}}}$ as a function of disorder strength *W* for *N* = 303 (empty red circles) and *N* = 610 (blue triangles) atoms. To illustrate the scale invariance of the system, the axes are rescaled by the zero-disorder ferromagnetic gap size *JN*. The markers represent the experimental data with statistical error bars, and the lines show the theoretical results obtained by exact diagonalization (see the ‘Numeric simulation of the large-detuning regime’ section). For **f**, data are presented as mean values ± s.e.m. Averages run over 290 measurements. For **g**, data are presented as mean values ± s.e.m. They are obtained from a fit of the polariton’s position with different atom numbers. The fitted data are averaged over 19 measurements. For **h**, data are obtained from a fit of the polariton’s position ± standard deviation, with different atom numbers. The fitted data are averaged over 290 measurements for *N* = 303 (empty red circles) and over 269 measurements for *N* = 610 (blue triangles).

In the central-mode model investigated so far, an essential role is played by the finite admixture of spin excitations to the delocalized photon field. For large detuning ${{{\varDelta }}}_{{{{\rm{ca}}}}}\gg g\sqrt{N}$, the cavity field is only virtually populated, giving rise to an all-to-all interaction between the spins, thereby realizing an effective LMG model^41–43^ (Fig. 3a and the ‘Effective model and atomic susceptibility in the large-detuning regime’ section). In the presence of a longitudinal random field, the Hamiltonian for these effective dynamics reads

2 $${\hat{H}}_{{{{\rm{LMG}}}}}=\mathop{\sum }\limits_{i=1}^{N}{\epsilon }_{i}\frac{{\hat{\sigma }}_{i}^{z}}{2}-JN{\hat{S}}^{+}{\hat{S}}^{-},$$

where *J* = *g*^2^/${{{\varDelta }}}_{{{{\rm{ca}}}}}$ is the strength of the spin-exchange interactions. Equation (2) is a particular case of the class of exactly solvable Richardson–Gaudin models^44,45^ that are ubiquitous in quantum many-body systems^32^.

Similar to the central-mode model, in the absence of disorder (*W* = 0), equation (2) describes the dynamics of a collective spin within the Hilbert subspace of symmetric states. The nonlinearity inherited from the spin–cavity coupling favours a ferromagnetic ground state, protected by a finite gap of size *JN*. A striking manifestation of ferromagnetism is the strong suppression of the zero-frequency magnetic response.

To realize the model of equation (2), we detune the cavity to the blue of the atomic transition by ${{{\varDelta }}}_{{{{\rm{ca}}}}}$/2π = 92 MHz, and probe the system at frequency *ω*_p_ in the vicinity of the bare atomic resonance *ω*_a_ (Fig. 3a). In this regime, the transmission of the cavity is negligible such that *χ*_p_ ≈ 0, and the atomic signal *χ*_a_(${{{\varDelta }}}_{{{{\rm{pa}}}}}$) (equation (15) provides the definition) directly measures the transverse spin susceptibility of the system at frequency ${{{\varDelta }}}_{{{{\rm{pa}}}}}$ = *ω*_p_ − (*ω*_a_ + 2*g*^2^/${{{\varDelta }}}_{{{{\rm{ca}}}}}$) (equation (12)). As shown in Fig. 3b,c, in the absence of disorder, the frequency dependence of *χ*_a_ reveals the finite ferromagnetic gap, with magnitude ${{{\varDelta }}}_{{{{\rm{FM}}}}}$, as well as the reduced zero-frequency susceptibility at ${{{\varDelta }}}_{{{{\rm{pa}}}}}$ = 0.

The signal is broadened by the finite decay rate of the excited atomic states, which reduces to a convolution of the response with the linewidth of the atomic transition (Supplementary Section 1.2).

We now investigate this model in the presence of disorder. Similar to the central-mode model, this breaks the description in terms of a collective spin, restoring the system’s ability to explore the full Hilbert space. For a given disorder strength *W*, the susceptibility (Fig. 3f) shows an asymmetric peak, corresponding to a collectively enhanced response superimposed with a weak and broad background whose width traces the disorder strength (Fig. 3b–e, dashed blue line). This is a manifestation of the gradual fragmentation of the collective spin, as disorder renders the individual spins off-resonant with each other. The peak is located at −${{{\varDelta }}}_{{{{\rm{FM}}}}}$, and we denote its amplitude by ${\chi }_{{{{\rm{a}}}}}^{{{{\rm{FM}}}}}$.

Tracking the location of this peak provides a measurement of the ferromagnetic gap as a function of *W*. Without disorder, this gap increases linearly with atom number (Fig. 3g). With increasing disorder, it decreases smoothly towards zero (Fig. 3h), where for low enough atom numbers, the gap is zero within our error bars. This demonstrates the competition between the infinite-range cavity-mediated interaction *J* and spectral disorder *W* for the dynamics of the effective model ${\hat{H}}_{{{{\rm{LMG}}}}}$.

Our results are in very good agreement with a simulation of the response *χ*_a_ of ${\hat{H}}_{{{{\rm{LMG}}}}}$ (see the ‘Numeric simulation of the large-detuning regime’ section), over the entire parameter regime (Fig. 3b–f): the simulated system sizes were set as the mean atom numbers *N* realized across all the experimental runs, and the effect of the atoms’ thermal motion on the atom–cavity coupling *g* has been taken into account, as in the near-resonant case. The decrease in the ferromagnetic gap (Fig. 3h) indicates a drastic change in the system properties as disorder increases. However, in the thermodynamic limit, the system is always ferromagnetic and no paramagnetic phase transition should occur. Indeed, intuitively, for any fixed disorder strength, increasing the number of atoms will always lead to an infinite number of close-to-resonance spins, enforcing ferromagnetism in the thermodynamic limit for an arbitrarily large disorder strength. However, for any finite number of atoms, there exists a disorder strength large enough to bring the ferromagnetic gap close to zero, by rendering each spin essentially spectrally isolated from all the others, thus crossing the system over into a paramagnet.

More precisely, our simulations show that finite systems display a minimal gap at disorder strength W* suggestive of critical behaviour; however, the value of W* diverges with increasing atom number (Supplementary Section 1.3 and Supplementary Fig. 2).

## Localization of excitations

The existence and distribution of energy resonances in disordered systems is the essence of Anderson localization. In our system, excitations can hop at arbitrarily large distances provided the spins are closely resonant. Disorder, thus, decimates the spins available for resonance by offsetting most spins from each other, but does not prevent long-distance propagation^33,35^.

Interestingly, although our spectroscopic probe does not yield spatially resolved information, it does carry relevant insights about the localization of excitations. Indeed, general arguments based on the hierarchy of Rényi entropies (see the ‘Participation ratio and its relation to susceptibility’ section) show that a system’s magnetic response may be used to bound the participation ratio of the excitations, that is, the number of spins contributing to the wavefunction. The participation ratio PR_1_ of the first excited state obeys

3 $${\chi }_{{{{\rm{a}}}},1}\ge {{{{\rm{PR}}}}}_{1}$$

at any *W* ≥ 0, where *χ*_a,1_ is the contribution of the first excited state to the atomic susceptibility when the system is probed on resonance with the transition to this state, from the global ground state (the ‘Participation ratio and its relation to susceptibility’ section provides the proof). The bound is reached for *W* = 0, where PR_1_ = *N* corresponds to a wavefunction uniformly distributed over all the spins, as well as in the limit *W* → ∞ in which the excitation becomes localized on a single spin (PR_1_ → 1). Our frequency-resolved measurement, thus, allows us to verify the fragmentation of the system’s collective excitations into ever-more localized wavefunctions, consistent with the expectations for eigenstates of the central-mode model^33,35,46^.

**Fig. 4:**
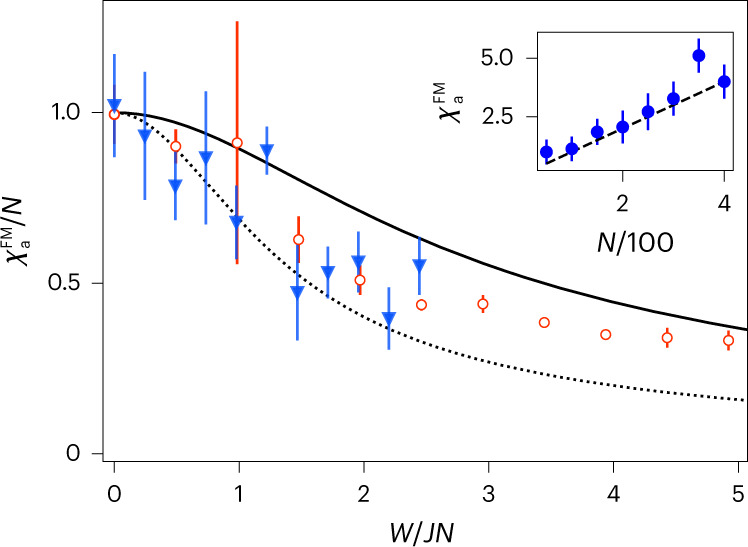
Participation-ratio bound from atomic susceptibility. Normalized atomic susceptibility ${\chi }_{{{{\rm{a}}}}}^{{{{\rm{FM}}}}}$, an upper bound to the participation ratio PR_1_ of the first excited state, for *N* = 303 (empty red circles) and *N* = 610 (blue triangles) as a function of normalized disorder strength. The solid black line shows the corresponding simulation results for *χ*_a,1_ of equation (3). The black dotted line is the directly simulated participation ratio of PR_1_. The inset shows the maximum value of the zero-disorder atomic susceptibility as a function of atom number, showing linear scaling expected from the definition of *χ*_a_ (equation (15)). Data are obtained from a fit of the polariton’s response ± standard deviation, with different atom numbers. The fitted data are averaged over 290 measurements for *N* = 303 (empty red circles) and over 269 measurements for *N* = 610 (blue triangles). For the inset, data are obtained from a fit of the polariton’s response ± standard deviation, with different atom numbers. The fitted data are averaged over 19 measurements.

Figure 4 shows the participation-ratio bound deduced from our measurements, showing a decrease by more than a factor of two as disorder reaches the largest values. On normalization of PR_1_ by the mean atom number *N* as well as *W* by the corresponding zero-disorder ferromagnetic gap *JN*, all the data collapse onto each other and agree with the simulations. The figure also shows the theoretically predicted value of PR_1_, which obeys the bound observed in the data.

Similar to the ferromagnetic gap, suggestive as these findings are, they do not herald a transition from delocalized to localized. For a fixed disorder strength, increasing the number of atoms leads to an infinite number of close-to-resonance spins at arbitrary distances, preventing full localization but leading to a semi-localized regime similar to the critical regime of the Anderson transition^33^.

## Conclusion

Our ability to introduce controlled disorder in cavity QED offers many timely and exciting prospects for further investigations, such as the study of Bardeen–Cooper–Schrieffer superconductivity^47,48^, where our atomic susceptibility measurements would directly map to the pairing gap. More broadly, equation (2) allows the direct simulation of Richardson–Gaudin models that are relevant to a variety of many-body systems, from superconductivity in ultrasmall grains to quark physics and neutron stars. Furthermore, the capabilities demonstrated in our experiment could also be used to study the effect of inhomogeneous broadening for quantum optics applications, particularly for superradiant laser clocks^49^. The combination of disorder with cavity-mediated interactions could further be used to study glassy phases of matter^50,51^.

Although the finite lifetime of the employed excited state limits the current investigations to one excitation above the fully polarized state, higher excitations can be probed by encoding the spins in the ground-state manifold and coupling them via Raman transitions^52^ or through the use of atoms with long-lived excited states^43^. Last, using high-resolution optics and time-resolved manipulation of the control light, it will become possible to program the otherwise homogeneous long-range cavity-mediated interaction in space and time, lifting one of the most stringent restrictions for the use of cavities in quantum simulation applications. In combination with small ultracold samples of our fermionic ^6^Li atoms, this will allow for the creation of random long-range interactions between fermionic degrees of freedom, one of the building blocks for holographic quantum matter^53^.

## Methods

### Experimental apparatus

The core of our setup is a high-finesse optical resonator placed inside an ultrahigh vacuum chamber^54^. The cavity has a finesse of 59 × 10^3^ and 13 × 10^3^ at 1,342 and 671 nm, respectively. The cavity is 25.9 mm long, 103 μm shorter than concentric, yielding a single-atom single-photon cooperativity of *η* = 6.4. The 1,342 nm light is used for frequency stabilization and dipole trapping and the 671 nm light allows for resonant coupling to the D2 transition of lithium.

We use in total two lasers, a 1,342 nm diode laser (main laser) that is Raman fibre amplified and then frequency doubled to generate light at 671 nm, and a laser diode emitting at 460 nm (light-shifting laser). The main laser is used for the magneto-optical trap, absorption imaging, cavity probing and trapping of the atoms in a cavity-enhanced optical dipole trap. It is stabilized to our cavity on the TEM_04_ mode at 1,342 nm. The length of the cavity itself can be controlled using piezoelectric actuators under the mirrors. We can stabilize the detuning between the D2 transition of lithium and the resonance frequency of our cavity in a large frequency range (>1 GHz), by using a sideband of the 671 nm beam sent to a saturated absorption spectroscopy cell. A feedforward scheme acting on both cavity and laser allows us to rapidly vary the cavity–atom detuning within the experimental sequence (maximum slew rate of 0.1 GHz ms^−1^) and holding the atoms in the cavity dipole trap. The light-shifting laser is stabilized using a commercial wavemeter.

### Preparation of atoms

We prepare an atomic cloud with a target atom number and size using a combination of laser cooling, spatial selection and cavity-assisted feedback techniques. We start by loading the atoms from a magneto-optical trap directly into the intracavity standing-wave dipole trap, with a temperature of about 200 μK and trap frequencies of *ω*_⊥_/2π = 22 kHz and *ω*_∥_/2π = 1.4 MHz in the transverse and longitudinal directions, respectively.

At this point, the cavity resonance frequency is set 1 GHz red-detuned with respect to the D2 transition. We then start an optical molasses phase using the magneto-optical trap beams and probing the cavity using light detuned by a fixed amount with respect to the resonance of the empty cavity. The dispersive shift in the cavity is reduced as atoms are lost during the molasses, until the probe becomes resonant with the cavity, leading to an increased transmitted photon flux detected by a single-photon counter. The molasses is stopped when the target atom number set by the predefined dispersive shift is reached and the sequence can continue. When turning off the optical molasses beams, we make sure that all the atoms are optically depumped into the $2{S}_{1/2}^{F = 1/2}$ manifold.

At this point of the sequence, the atomic cloud measures a length of 0.5 mm, populating about 750 pancakes, each containing between 0.4 and 4.0 atoms on average. We empty all but the central 180 sites using radiation pressure, by imaging an opaque mask on the centre of the cloud with a laser resonant on the D2 transitions (Extended Data Fig. 1a). We then shift the cavity on resonance within 30 ms with the $2{S}_{1/2}^{F = 3/2}$–2*P*_3/2_ transition, leading to a detuning of 228 MHz (hyperfine splitting of ^6^Li) with respect to the $2{S}_{1/2}^{F = 1/2}$–2*P*_3/2_ transition resonant with the atoms.

We then perform fast cavity transmission spectroscopy by sweeping a weak probe over the cavity resonance. The dispersive shift in the cavity is used to extract the initial number of atoms in the *F* = 1/2 state. A similar sweep is performed after the interrogation of the disordered system. Together, they allow for the characterization of probe-induced atom losses on a shot-to-shot basis.

The deterministic preparation suppresses drifts in the atom number and allows for long-term averages of the experimental results. However, we still observe shot-to-shot fluctuations in the atom number that stem from the Poissonian nature of the trap loading and losses and the atom removal procedure described above. The standard deviation of these fluctuations is about 35 and 60 atoms for the data presented in Figs. 2 and 3, respectively.

### Implementation of disorder

We encode the two-level system using the $2{S}_{1/2}^{F = 1/2}$ (|*g*〉) and 2*P*_3/2_ (|*e*〉) states of our ^6^Li atoms. The transition frequency of the atoms can be tuned by light shifting the excited state |*e*〉. In particular, this is achieved by dressing the 2*P*_3/2_ state with the higher-lying 4*D*_5/2_ manifold using a control laser at 460 nm detuned from resonance (Fig. 1c) by ${{{\varDelta }}}_{{{{\rm{blue}}}}}$. We first calibrated the light shift of the excited state—due to a single Gaussian beam with a waist of 120 μm at ${{{\varDelta }}}_{{{{\rm{blue}}}}}$ = 50 MHz—by performing absorption spectroscopy of the D2 transition, similar to another work^55^. Taking the absorption images of the cloud at different imaging frequencies, we reconstructed the spatial distribution of the light shift of one of the two identical beams that generate the light-shifting lattice when sent together (Extended Data Fig. 1d). We performed this spectroscopy both in situ and after releasing the atoms from the cavity dipole trap, allowing us to measure the trap-related shift of the 2*P*_3/2_–4*D*_5/2_ transition to be 90 MHz.

Furthermore, we characterized the dependence of the cavity transmission spectrum on the detuning of the light-shifting laser, showing an avoided crossing for both states of the Autler–Townes doublet, particularly the light-shifted single-photon 2*S*_1/2_–2*P*_3/2_ transition and two-photon 2*S*_1/2_–4*D*_5/2_ transition (Extended Data Fig. 1e). We observed increased atom losses for small detunings of the light-shifting laser, pointing towards radiation-pressure-induced atom losses, occurring when atoms are promoted to the 2*P*_3/2_ state during the spectroscopic measurements. We minimized this effect by choosing the maximal detuning (400 MHz blue detuned from the 2*P*_3/2_–4*D*_5/2_ transition), allowing us to go up to *W* = 26 MHz for the maximum available power of the laser of 7.3 mW per lattice beam.

The light-shifting laser produces a dipole potential on the atoms in the ground state of about 5 × 10^−6^ smaller than the light shift of the 2*P*_3/2_ state, negligible compared with the intracavity trapping potential.

Both light-shifting lattice beams are linearly polarized perpendicular to the cavity axis, and set the direction of the quantization axis. We then probe the cavity using π-polarized light, to avoid any vector light-shift effect of the light-shifting beam. Because atoms reside in the *F* = 1/2 hyperfine manifold, the π transition used for cavity interrogation is free of tensor light-shift effects. As a result, even though our sample comprises an incoherent mixture of the two magnetic sublevels of the *F* = 1/2 manifold, the two components experience a strictly identical light shift and probe beam, contributing equally to the signal without further broadening effects. Cross-optical pumping between the two does not deteriorate the signal in the linear-response regime explored in this work.

### Interrogation

Once the preparation phase is completed, we tune the cavity to the desired length and illuminate the cloud with the light-shifting lattice. We send a cavity probe pulse with a duration of 5 or 60 μs for the measurements presented in Figs. 2 and 3, respectively. During this measurement, we monitor the photons leaking out of the cavity using a single-photon counter, to infer the optical response. At zero magnetic field, the transition between |*g*〉 and |*e*〉 is not closed, and an atom in the 2*P*_3/2_ state can decay into the *F* = 3/2 ground-state manifold, denoted as an auxiliary state |*a*〉. This state is not coupled to the cavity field, owing to the large hyperfine splitting. Since the decay can only happen from state |*e*〉, the population accumulated in the *F* = 3/2 state is directly proportional to the excited-state population $\langle {\hat{S}}_{z}\rangle$ integrated over the probe-pulse duration.

The population of the *F* = 3/2 state is measured after the interrogation of the disordered system using a cavity transmission spectroscopy, with the cavity tuned on resonance with the *F* = 3/2 to 2*P*_3/2_ transition (Extended Data Fig. 2b, right). In this configuration, the power of the cavity transmission is suppressed by 1/(1 + *η*)^2^ in the presence of a single atom in the *F* = 3/2 state, yielding a single-atom-level sensitivity for the detection of atomic response.

In practice, we implement the detection by sweeping the frequency of the on-axis probe over the cavity resonance, yielding an average photon count of four photons per sweep for the empty |*a*〉 manifold (Extended Data Fig. 2c (inset), green histogram). The frequency sweep is essential since it removes the systematic effects of dispersive shifts on the depumping detection resulting from the presence of atoms in the |*g*〉 state. Extended Data Fig. 2c shows the dependence of the number of transmitted photons on the laser power during interrogation, showing the expected exponential trend (see the ‘Measurement of atomic susceptibility’ section), allowing for the characterization of atomic susceptibility. At large probe powers, we observe a deviation from the exponential model that is due to saturation effects and atom losses. The data presented in this work were measured at different probe powers, and the measurements with an average photon count below 1.5 photons per sweep were neglected (Extended Data Fig. 2c, dashed line), ensuring that no additional broadening of the resonances is introduced.

### Susceptibility in the near-resonant regime

In this section, we provide some details on the calculations of the susceptibility in the near-resonant regime, whose results are presented in the ‘Near-resonant regime and grey states’ section.

In our calculations, we account for fluctuations in both atom number *N* and atom–cavity couplings *g*. Specifically, we average the susceptibility over 1,000 different values of *N* randomly sampled from a normal distribution. The mean and standard deviation of the *N* distribution have been determined by fitting the experimental data at *W* = 0. For each value of *N*, we consider a generalized version of the Tavis–Cummings Hamiltonian in equation (1), namely,

4 $${\hat{H}}_{{{{\rm{TCr}}}}}={{{\varDelta }}}_{{{{\rm{ca}}}}}{\hat{a}}^{{\dagger} }\hat{a}+\mathop{\sum }\limits_{i=1}^{N}{g}_{i}\left({\hat{\sigma }}_{i}^{+}\,\hat{a}+{\hat{\sigma }}_{i}^{-}\,{\hat{a}}^{{\dagger} }\right)+\mathop{\sum }\limits_{i=1}^{N}{\epsilon }_{i}\,\frac{{\hat{\sigma }}_{i}^{z}}{2}\,,$$

where couplings *g*_*i*_ are randomly generated to account for the finite temperature of atoms and the polarization of probe light. Specifically, the *g*_*i*_ values are proportional to the square root of the cavity-field intensity at the atom positions, which are extracted from a thermal distribution (the ‘Preparation of atoms’ section provides the parameters). We also account for the fact that the *N* atoms are randomly distributed across ~100 pancakes: to this end, we randomly select *N* site energies among the set ${\epsilon }_{i}\in \{\frac{W}{2}\cos (2\uppi {\lambda }_{\mathrm{l}}j/{\lambda }_{\mathrm{s}})\,,\,j=1,\ldots ,100\}$, where *λ*_l_ = 671 nm is the lattice wavelength and *λ*_s_ = 1,040 nm is the light-shift wavelength.

For each value of *N*, as per other work^35,56^, we employ a Green function formalism in the linear-response regime. In such a situation, the cavity susceptibility (cavity transmission) at a given probe–cavity detuning ${{{\varDelta }}}_{{{{\rm{pc}}}}}$ is

5 $${\chi }_{{{{\rm{p}}}}}({{{\varDelta }}}_{{{{\rm{pc}}}}})\propto -{{{\rm{Im}}}}\left(\left\langle G\right\vert \hat{a}\frac{1}{{{{\varDelta }}}_{{{{\rm{pc}}}}}-\hat{{{{\mathcal{H}}}}}}{\hat{a}}^{{\dagger} }\left\vert G\right\rangle \right)\,,$$

where |*G*〉 is the ground state. In equation (5), we introduced the non-Hermitian Hamiltonian as

6 $$\hat{{{{\mathcal{H}}}}}={\hat{H}}_{{{{\rm{TCr}}}}}-i\frac{{{\varGamma }}}{2}\mathop{\sum }\limits_{i=1}^{N}{\hat{\sigma }}_{i}^{+}{\hat{\sigma }}_{i}^{-}-i\frac{\kappa }{2}{\hat{a}}^{{\dagger} }\hat{a}\,,$$

which includes the generalized Tavis–Cummings Hamiltonian (equation (4)) and two terms describing cavity losses and atom decay, respectively. Similarly, the atomic susceptibility is computed by summing the transition probabilities to all the atomic states ${\hat{\sigma }}_{i}^{+}\left\vert G\right\rangle$, namely,

7 $${\chi }_{{{{\rm{a}}}}}({{{\varDelta }}}_{{{{\rm{pc}}}}})\propto \mathop{\sum }\limits_{i=1}^{N}{\left\vert \left\langle G\right\vert {\hat{\sigma }}_{i}^{-}\frac{1}{{{{\varDelta }}}_{{{{\rm{pc}}}}}-\hat{{{{\mathcal{H}}}}}}{\hat{a}}^{{\dagger} }\left\vert G\right\rangle \right\vert }^{2}\,.$$

### Measurement of atomic susceptibility

We now show that the atomic susceptibility *χ*_a_(${{{\varDelta }}}_{{{{\rm{pa}}}}}$) (see the ‘Large-detuning regime and LMG model’ section), can be extracted from the measurements of atomic population *P*_A_(*t*) of the auxiliary state |*a*〉 at a given point in time *t*. Intuitively, it is plausible that *χ*_a_(${{{\varDelta }}}_{{{{\rm{pa}}}}}$) and *P*_A_(*t*) should be connected: on one hand, *χ*_a_(${{{\varDelta }}}_{{{{\rm{pa}}}}}$) is simply a rescaling of the absorptive part of the dynamic susceptibility *χ*″(${{{\varDelta }}}_{{{{\rm{pa}}}}}$) (equations (13)–(15)) of the effective model described by equation (11) and thus quantifies the time-averaged energy absorbed by this system when subjected to a perturbation at frequency ${{{\varDelta }}}_{{{{\rm{pa}}}}}$. On the other hand, the system can absorb energy from the probe beam only via coherent excitations of the atomic population from state |*g*〉 ($2{S}_{1/2}^{F = 1/2}$) to |*e*〉 (2*P*_3/2_). The population of state |*a*〉 ($2{S}_{1/2}^{F = 3/2}$) can then change only via spontaneous decay from state |*e*〉 at rate *Γ*_a_. Therefore, detecting *P*_A_(*t*) > 0 implies that the system has absorbed energy via atomic excitations. Furthermore, the probability to excite the system into a collective state containing an atomic excitation on probing is maximized when the probe frequency ${{{\varDelta }}}_{{{{\rm{pa}}}}}$ is resonant with transitions from the system’s collective ground state. It follows that the total atomic population *P*_A_(*t*_meas_) found in state |*a*〉 after the interrogation time is a measure of how susceptible the effective model was to excitations introduced by the probe at frequency ${{{\varDelta }}}_{{{{\rm{pa}}}}}$.

We give the above intuition an analytic foundation by modelling the experimental sequence (see the ‘Preparation of atoms’, ‘Implementation of disorder’ and ‘Interrogation’ sections) via a Lindblad master equation (Supplementary Section 1.2 provides details of the derivation). We derive an equation of motion for *P*_A_(*t*) in terms of *χ*_a_(${{{\varDelta }}}_{{{{\rm{pa}}}}}$) using the fact that the probe beam’s amplitude *Ω*_p_ is much weaker than the natural linewidth *Γ* of ^6^Li, that is, that atomic excitations decay at a rate much faster than the rate at which they are introduced by the probe beam, namely, |*Ω*_p_| ≪ *Γ*. This yields the relation

8 $${P}_{{{{\rm{A}}}}}({t}_{{{{\rm{meas}}}}})=1-\exp \left(-\frac{{{{\varGamma }}}_{{{{\rm{a}}}}}}{{({{\varGamma }}/2)}^{2}}{\left\vert \frac{g{{{\varOmega }}}_{{{{\rm{p}}}}}}{{{{\varDelta }}}_{{{{\rm{ca}}}}}}\right\vert }^{2}{\chi }_{{{{\rm{a}}}}}({{{\varDelta }}}_{{{{\rm{pa}}}}}){t}_{{{{\rm{meas}}}}}\right),$$

evaluated here at the measurement time *t* = *t*_meas_. This result confirms the monotonic relation between *P*_A_(*t*) and *χ*_a_(${{{\varDelta }}}_{{{{\rm{pa}}}}}$). It is obtained with respect to the experiment’s initial conditions *p*_*G*_(0) = 1 and *P*_A_(0) = 0, and is valid for times *t* ≫ (*Γ*/2)^−1^.

The saturation of *P*_A_(*t*_meas_) as a function of probe power |*Ω*_p_|^2^ (Extended Data Fig. 2c) is captured by equation (8). Further, for a given probe power, the saturation rate is maximal at those probe frequencies ${{{\varDelta }}}_{{{{\rm{pa}}}}}$ at which *χ*_a_(${{{\varDelta }}}_{{{{\rm{pa}}}}}$) is the largest: since population transfer from |*G*〉 to state |*m*〉 of the single-excitation manifold (SEM) (see the ‘Effective model and atomic susceptibility in the large-detuning regime’ section) is maximized when the probe frequency is resonant with the transition frequency *E*_*mG*_ (that is, resonant with a frequency at which the system is most susceptible to perturbations, as quantified by *χ*_a_(${{{\varDelta }}}_{{{{\rm{pa}}}}}$)), the concomitant accumulation of population in the auxiliary state is also maximized. Conversely, for a fixed measurement time *t*_meas_, saturation of the signal *P*_A_(*t*_meas_) can be suppressed by reducing the probe’s power. This is crucial for the precision of the experimental data presented in Fig. 3 (see the ‘Interrogation’ section) where the experimental technique for measuring *P*_A_(*t*_meas_) is discussed.

### Effective model and atomic susceptibility in the large-detuning regime

In this section, we demonstrate that the dynamics of our system are described by the effective Hamiltonian of equation (2) when the cavity is tuned far into the dispersive regime, such that ${{{\varDelta }}}_{{{{\rm{ca}}}}}$ ≡ *ω*_c_ − *ω*_a_ is the dominant energy scale.

Our starting point is the disordered Tavis–Cummings Hamiltonian ${\hat{H}}_{{{{\rm{TC}}}}}$, which is expressed in equation (1) relative to the rotating frame of the bare atomic resonance frequency *ω*_a_. Within this rotating frame, the probe beam is described by the perturbation $\hat{V}(t)={{{\varOmega }}}_{{{{\rm{p}}}}}{\mathrm{e}}^{-\mathrm{i}({\omega }_{{{{\rm{p}}}}}-{\omega }_{{{{\rm{a}}}}})t}{\hat{a}}^{{\dagger} }+{{{\rm{h.c.}}}}$, with probe-laser and Rabi frequency *ω*_p_ and *Ω*_p_, respectively.

The total Hamiltonian is ${\hat{H}}_{{{{\rm{TC}}}}}+\hat{V}(t)$, and thus, the equation of motion of the (Heisenberg picture) photonic operator $\hat{a}(t)$ is

9 $${\partial }_{t}\hat{a}(t)=-i\left[\hat{a}(t),{\hat{H}}_{{{{\rm{TC}}}}}+\hat{V}(t)\right]-(\kappa /2)\hat{a}(t),$$

where the last term takes into account cavity losses. Using the fact that these are sub-dominant, that is, ${{{\varDelta }}}_{{{{\rm{ca}}}}}$ ≫ *κ* (see the ‘Effective model and atomic susceptibility in the large-detuning regime’ and ‘Large-detuning regime and LMG model’ sections), the cavity mode adiabatically follows the evolution of the spin degrees of freedom as

10 $$\hat{a}(t)=-\frac{g\sqrt{N}{\hat{S}}^{-}+{{{\varOmega }}}_{{{{\rm{p}}}}}{\mathrm{e}}^{-\mathrm{i}({\omega }_{{{{\rm{p}}}}}-{\omega }_{{{{\rm{a}}}}})t}}{{{{\varDelta }}}_{{{{\rm{ca}}}}}}.$$

Substituting this expression into ${\hat{H}}_{{{{\rm{TC}}}}}+\hat{V}(t)\,$ eliminates the cavity mode, and one obtains (up to an irrelevant constant term) the effective spin Hamiltonian as

11 $$\hat{H}(t)={\hat{H}}_{{{{\rm{LMG}}}}}-\hat{{{{\mathcal{V}}}}}(t)\,,\quad {{{\rm{where}}}}$$

12 $$\hat{{{{\mathcal{V}}}}}(t)=\frac{g\sqrt{N}}{{{{\varDelta }}}_{{{{\rm{ca}}}}}}\left({{{\varOmega }}}_{{{{\rm{p}}}}}{\mathrm{e}}^{-\mathrm{i}{{{\varDelta }}}_{{{{\rm{pa}}}}}t}{\hat{S}}^{+}+{{{\rm{h.c.}}}}\right),$$

with ${{{\varDelta }}}_{{{{\rm{pa}}}}}$ ≡ *ω*_p_ − (*ω*_a_ + 2*g*^2^/${{{\varDelta }}}_{{{{\rm{ca}}}}}$) and ${\hat{H}}_{{{{\rm{LMG}}}}}$ is as per equation (2). We note that the above equations are obtained after performing an additional rotating-frame transformation, which serves only to remove an otherwise constant contribution of $(2{g}^{2}N/{{{\varDelta }}}_{{{{\rm{ca}}}}}){\hat{S}}^{z}$ to equation (2).

Having obtained the above effective model, we now derive the form of atomic susceptibility *χ*_a_(${{{\varDelta }}}_{{{{\rm{pa}}}}}$) in the dispersive regime. In particular, *χ*_a_(${{{\varDelta }}}_{{{{\rm{pa}}}}}$) is obtained from the absorptive part *χ*″(${{{\varDelta }}}_{{{{\rm{pa}}}}}$) of the dynamic susceptibility of effective model ${\hat{H}}_{{{{\rm{LMG}}}}}$ of equation (2), when the latter is initialized in its ground state $\left\vert G\right\rangle {\equiv \bigotimes }_{i = 1}^{N}{\left\vert g\right\rangle }_{i}\,$ and subsequently subjected to the probe via the interaction $\hat{{{{\mathcal{V}}}}}(t)$ of equation (12). Studying the dynamic susceptibility is motivated by the fact that the probe beam is weak (|*Ω*_p_| ≪ *Γ*), so that one may treat $\hat{{{{\mathcal{V}}}}}(t)$ as a perturbation within the regime of linear response^57^. In particular, |*Ω*_p_| ≪ *Γ* implies that atomic excitations decay much faster than the rate at which they are introduced into the system, so that one may study the limit in which there is at most a single excitation present in the system. That is, one needs to only consider the eigenstates |*G*〉 and ${\{\left\vert m\right\rangle \}}_{m = 1}^{N}$, where the latter set of states forms the SEM of ${\hat{H}}_{{{{\rm{LMG}}}}}$. We denote the respective eigenenergies as ${{{{\mathcal{E}}}}}_{G},{{{{\mathcal{E}}}}}_{m}$, and the spectral gaps as ${E}_{mG}\equiv {{{{\mathcal{E}}}}}_{m}-{{{{\mathcal{E}}}}}_{G}$, for *m* = 1, …, *N*. With respect to this basis, we then have

13 $${\chi }^{{{\prime\prime}} }({{{\varDelta }}}_{{{{\rm{pa}}}}})=\uppi \mathop{\sum}\limits_{m\in {{{\rm{SEM}}}}}{\left\vert \left\langle m\right\vert \frac{g\sqrt{N}{{{\varOmega }}}_{{{{\rm{p}}}}}}{{{{\varDelta }}}_{{{{\rm{ca}}}}}}{\hat{S}}^{+}\left\vert G\right\rangle\right\vert }^{2}\delta ({{{\varDelta }}}_{{{{\rm{pa}}}}}-{E}_{mG}).$$

In what follows, we approximate the Dirac delta functions in equation (13) as Lorentzian responses:

14 $${\delta }_{\gamma }(\omega )\equiv \frac{\gamma /\uppi }{{\gamma }^{2}+{\omega }^{2}}\,{{{\rm{such}}}}\,{{{\rm{that}}}}\,\delta (\omega )=\mathop{\lim }\limits_{\gamma \to 0}{\delta }_{\gamma }(\omega ).$$

Here *γ* is the linewidth of the (normalized) resonance, which according to the Wiener–Khintchine theorem^58,59^ corresponds to a finite experimental measurement time 1/*γ*. The dimensionless atomic susceptibility *χ*_a_(${{{\varDelta }}}_{{{{\rm{pa}}}}}$) (Fig. 3) is then finally obtained from *χ*″(${{{\varDelta }}}_{{{{\rm{pa}}}}}$) as

15 $$\begin{array}{l}{\chi }_{{{{\rm{a}}}}}({{{\varDelta }}}_{{{{\rm{pa}}}}})\,=\,\gamma {\left\vert \frac{{{{\varDelta }}}_{{{{\rm{ca}}}}}}{g{{{\varOmega }}}_{{{{\rm{p}}}}}}\right\vert }^{2}{\chi }^{{{\prime\prime}} }({{{\varDelta }}}_{{{{\rm{pa}}}}})\\ \qquad \qquad=\mathop{\sum}\limits_{m\in {{{\rm{SEM}}}}}N{\left\vert \left\langle m\right\vert {\hat{S}}^{+}\left\vert G\right\rangle\right\vert }^{2}\frac{{\gamma }^{2}}{{\gamma }^{2}+{({{{\varDelta }}}_{{{{\rm{pa}}}}}-{E}_{mG})}^{2}}\\\qquad \qquad \equiv \mathop{\sum}\limits_{m\in {{{\rm{SEM}}}}}{\chi }_{{{{\rm{a}}}},m}({{{\varDelta }}}_{{{{\rm{pa}}}}}).\end{array}$$

At zero disorder, only the first excited state $\left\vert m=1\right\rangle ={\hat{S}}^{+}\left\vert G\right\rangle\,$ contributes, such that on resonance, *χ*_a_(${{{\varDelta }}}_{{{{\rm{pa}}}}}$ = *E*_1*G*_) = *χ*_a,1_(*E*_1*G*_) = *N* (Fig. 4, inset).

### Participation ratio and its relation to susceptibility

Here we prove the relation, given in inequality (3), between the atomic susceptibility and participation ratio. The participation ratio quantifies the extent to which a given state is (de)localized over a basis of interest. In our context, we wish to study the (de)localization of an SEM eigenstate $\left\vert m\right\rangle \equiv \mathop{\sum }\nolimits_{i = 1}^{N}{c}_{mi}{\hat{\sigma }}_{i}^{+}\left\vert G\right\rangle$ of the LMG Hamiltonian of equation (2) over the spins *i* of the system. This is quantified by the participation ratio as

16 $${{{{\rm{PR}}}}}_{m}={\left(\mathop{\sum }\limits_{i = 1}^{N}| {c}_{mi}{| }^{4}\right)}^{-1}\in [1,N],$$

of which the limiting values 1 and *N* are respectively obtained at ${c}_{mi}={\delta }_{i,{i}^{* }}$ (full localization at some site i*, achieved at *W* → ∞), and ${c}_{mi}=\frac{1}{\sqrt{N}},\,\forall \,i$ (full delocalization over all *N* sites, achieved at *W* → 0).

Our proof of inequality (3) relies on the identification of PR_1_ and *χ*_a,1_(*E*_1*G*_) (equation (17)) as monotonic functions of different Rényi entropies ${H}_{\alpha }(\overrightarrow{p})=\frac{1}{1-\alpha }\log ({\sum }_{i}{p}_{i}^{\alpha })$, and then exploiting the hierarchy ${H}_{{\alpha }_{1}}(\overrightarrow{p})\ge {H}_{{\alpha }_{2}}(\overrightarrow{p})$ for any real numbers *α*_2_ ≥ *α*_1_ ≥ 0 (ref. ^60^). To this end, we note that (1) on resonance ${{{\varDelta }}}_{{{{\rm{pa}}}}}$ = *E*_*mG*_, the *m*th summand of the atomic susceptibility defined in equation (15) reduces to

17 $$\qquad \,{\chi }_{{{{\rm{a}}}},m}({E}_{mG})={\left\vert \mathop{\sum }\limits_{i = 1}^{N}{c}_{mi}\right\vert }^{2}\in [1,N],$$

whose limiting values are obtained with the same distributions of *c*_*mi*_ as for PR_*m*_ (equation (16)). (2) Using Perron–Frobenius theory^61^, one can show that the lowest SEM eigenstate |*m* = 1〉 of ${\hat{H}}_{{{{\rm{LMG}}}}}$ (as defined in equation (2)) satisfies *c*_1*i*_ ≥ 0, ∀ *i* = 1, …, *N*. Hence, one has that ${c}_{1i}=+\sqrt{{p}_{1i}}$, where *p*_*mi*_ ≡ |*c*_*mi*_|^2^ are the probabilities associated to the amplitudes *c*_*mi*_.

Now, for the identification with Rényi entropies, we expand both sides of inequality (3) and employ point (2) above. This yields

18 $$\begin{array}{ll}&{\chi }_{{{{\rm{a}}}},1}({E}_{1G})={\left\vert \mathop{\sum}\limits_{i}{c}_{1i}\right\vert }^{2}={\left(\mathop{\sum}\limits_{i}{p}_{1i}^{{\alpha }_{1}}\right)}^{\frac{1}{1-{\alpha }_{1}}}=\exp ({H}_{{\alpha }_{1}}(\overrightarrow{{p}_{1}})),\\ &{{{{\rm{PR}}}}}_{1}={\left(\mathop{\sum }\limits_{i = 1}^{N}| {c}_{1i}{| }^{4}\right)}^{-1}={\left(\mathop{\sum}\limits_{i}{p}_{1i}^{{\alpha }_{2}}\right)}^{\frac{1}{1-{\alpha }_{2}}}=\exp ({H}_{{\alpha }_{2}}(\overrightarrow{{p}_{1}})),\end{array}$$

where *α*_1_ = 1/2, *α*_2_ = 2 and ${\overrightarrow{p}}_{1}\equiv ({p}_{11},{p}_{12},\ldots ,{p}_{1N})\,$. Since exp(*x*) is monotonic, the hierarchy of Rényi entropies is preserved and thus

19 $$\exp ({H}_{{\alpha }_{1}}(\,{\overrightarrow{p}}_{1}))\ge \exp ({H}_{{\alpha }_{2}}(\,{\overrightarrow{p}}_{1}))\,{{{\rm{for}}}}\,{\alpha }_{1}=1/2\,{{{\rm{and}}}}\,{\alpha }_{2}=2.$$

This concludes the proof.

We briefly comment on how the participation-ratio bound of inequality (3) may be measured: due to the finite atomic linewidth (see the ‘Measurement of atomic susceptibility’ section), extracting only the *m* = 1 summand of the atomic susceptibility is not feasible, as nearby resonances will add to the measured signal. What can be feasibly extracted is the amplitude ${\chi }_{{{{\rm{a}}}}}^{{{{\rm{FM}}}}}$ of the full susceptibility of equation (15), which satisfies ${\chi }_{{{{\rm{a}}}}}^{{{{\rm{FM}}}}}\equiv {\chi }_{{{{\rm{a}}}}}({E}_{1G})\ge {\chi }_{{{{\rm{a}}}},1}({E}_{1G})$, by definition. This is the data shown in Fig. 4.

In closing, we note that inequality (3) (as well as its looser form in terms of the full *χ*_a_(*E*_1*G*_)) becomes an equality in both limits of |*m* = 1〉 being fully (de)localized. This, too, follows from the above expression in terms of Rényi entropies: for all *α* ≥ 0, ${H}_{\alpha }(\overrightarrow{p})=\log (N)$ if *p*_*i*_ = 1/*N*, ∀ *i* = 1, …, *N* (maximal uncertainty), and ${H}_{\alpha }(\overrightarrow{p})=0$ if ${p}_{i}={\delta }_{i,{i}^{* }}$ for some i* = 1, …, *N* (maximal certainty).

The above discussion exemplifies that the participation ratio is an entropic measure, quantifying the degree of (un)certainty ((de)localization)—obtained from some state’s expansion coefficients—as to its spread over a chosen set of degrees of freedom (basis). In fact, for any basis {|*i*〉}, the generalized inverse participation ratio IPR_*q*_(|*ψ*〉) is related to Rényi entropies via its multifractal dimension *D*_*q*_: combining equations (2) and (3) in Supplementary Information, one has ${D}_{q}=\frac{d}{1-q}\frac{\log ({{{{\rm{IPR}}}}}_{q}(\left\vert \psi \right\rangle ))}{\log (N)}$. As in the above discussion, this can be expressed in terms of Rényi entropies as ${D}_{q}=d\frac{{H}_{\alpha = q}(\overrightarrow{p})}{\log (N)}$, where $\overrightarrow{p}=({\left\vert \langle i = 1| \psi \rangle \right\vert }^{2},\ldots ,{\left\vert \langle i = N| \psi \rangle \right\vert }^{2})$. This relation exemplifies the intimate link between entropy and quantifiers of a state’s (de)localization properties, and has as an immediate consequence that *D*_*q*_ decays monotonically with *q* ≥ 0.

### Numeric simulation of the large-detuning regime

We compute *χ*_a_ and participation ratios by diagonalizing the random LMG Hamiltonian of equation (2) for system sizes *N* = 303 and 610. These system sizes correspond to the mean atom numbers realized in the experiment, which were determined from the dispersive shift *JN* = *g*^2^*N*/${{{\varDelta }}}_{{{{\rm{ca}}}}}$ measured at zero disorder (*W* = 0), for each iteration of the measurement sequence (see the ‘Preparation of atoms’ section). The effect of the atoms’ thermal motion on the value of *g* was taken into account for the conversion of the dispersive shifts into atom numbers, as well as for the matrix elements of the Hamiltonian. Taking the mean atom number across all the experimental runs yields the system sizes quoted above.

We choose the random energy shifts *ϵ*_*i*_ in two different ways: (1) from the incommensurate light-shift potential generating correlated quasi-random disorder (as discussed in the main text) and (2) independent and identically distributed *ρ*_a_. For both cases, we find quantitative agreement of *χ*_a_ as well as the participation ratio, within numerical accuracy.

The Hamiltonian matrix is constructed with respect to the basis states $\left\vert i\right\rangle ={\hat{\sigma }}_{i}^{+}\left\vert G\right\rangle$ of the SEM, and diagonalized exactly. In the absence of disorder, that is, *ϵ*_*i*_ = 0 ∀ *i*, the diagonalization is analytically tractable, and the eigenenergies are ${{{{\mathcal{E}}}}}_{1}=-NJ/2$ and ${{{{\mathcal{E}}}}}_{m}=NJ/2$ for *m* = 2,…, *N*. Consequently, the zero-disorder ferromagnetic gap ${{{\varDelta }}}_{{{{\rm{FM}}}}}\equiv {{{{\mathcal{E}}}}}_{2}-{{{{\mathcal{E}}}}}_{1}=JN$, as mentioned in the main text. However, the presence of disorder mixes the Hamiltonian’s zero-disorder eigenstates, necessitating the analysis through numerical diagonalization. Using the numerically determined eigenenergies and eigenstates, we compute the atomic susceptibility and participation ratio using equations (15) and (16), respectively. We average these quantities with respect to 2,000 disorder realizations of the Hamiltonian, the results of which are illustrated in Figs. 3 and 4. The corresponding variances are strongly suppressed, falling within the linewidths of the simulated data.

## Online content

Any methods, additional references, Nature Portfolio reporting summaries, source data, extended data, supplementary information, acknowledgements, peer review information; details of author contributions and competing interests; and statements of data and code availability are available at 10.1038/s41567-023-02033-3.

## Supplementary information

Supplementary Information Supplementary Section 1, Figs. 1 and 2 and references.

## Data Availability

The datasets generated and analysed in the current study are available via Zenodo at 10.5281/zenodo.7074544 (ref. ^62^).

## Code availability

The codes used for the analysis of the experimental data and for simulations are available from the corresponding author upon reasonable request.
