# Supplementary material for: Engineering random spin models with atoms in a high-finesse cavity
Source: Nat Phys. 2023 May 4;19(8):1128–34. doi: 10.1038/s41567-023-02033-3 (PMC10415180; doi:10.1038/s41567-023-02033-3)
Supplement: Supplementary file 1 — Supplementary Section 1, Figs. 1 and 2 and references. [file 41567_2023_2033_MOESM1_ESM.pdf]

nature physics

Article

https://doi.org/10.1038/s41567-023-02033-3

Engineering random spin models with atoms in a high-finesse cavity

In the format provided by the authors and unedited

1 Supplementary Information

1.1 Structure of the eigenstates in the near-resonant regime: multifractality and quasi-random potential

In this section, we discuss the structure of the eigenstates of the TC Hamiltonian Eq. (1) in the near-resonant regime ($\Delta_{\rm ca} = 0$). As recently found in Ref. [1], the eigenstates of the TC Hamiltonian with random uniformly distributed atomic energies are always multifractal, for any non-vanishing disorder strength. Here, we show that the same result is obtained with a correlated energy potential,

$$\epsilon_i = \frac{W}{2}\cos(2\pi Q i), \tag{S1}$$

which is incommensurate to the lattice spacing [here we choose $Q = (\sqrt{5} - 1)/2$]. This energy potential, in the presence of nearest-neighbour hopping and in the absence of a cavity mode, constitutes the well-studied Aubry–André–Harper (AAH) model, which has a localisation–delocalisation transition in the thermodynamic limit [2, 3]. This is in contrast to the one-dimensional Anderson model, characterised by a random uniformly distributed energy potential with nearest-neighbour hopping, which has no transition and is always localised in the $N \to \infty$ limit [4]. Moreover, the correlated potential in Eq. (S1) with power-law hopping has a rich phase diagram, including localised, delocalised and multifractal phases, with mobility edges [5]. Therefore, it is important to check whether this energy potential affects the results of Ref. [1] regarding the multifractality of the TC Hamiltonian eigenstates.

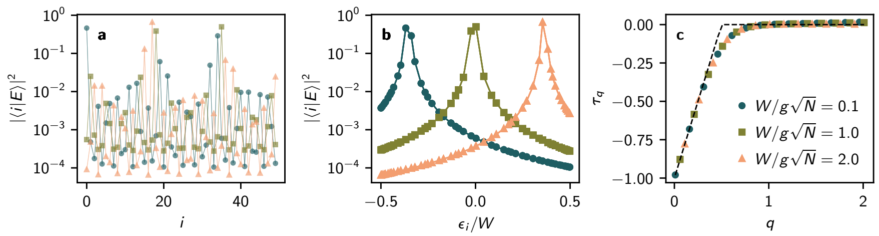

SUPPLEMENTARY FIG. S1 Multifractality of the eigenstates of the TC Hamiltonian near resonance. a, Squared amplitudes of a representative grey-state eigenfunction of the TC Hamiltonian Eq. (1) for $\Delta_{\rm ca} = 0$ on the atoms $|i\rangle = \hat{\sigma}_i^+ |G\rangle$, as a function of the atom index i. Here, $N = 50$ and three different disorder strengths have been considered (see legend in panel **c**). **b**, Squared amplitudes of the same eigenfunctions shown in panel **a**, here as a function of the atomic energies ϵ_i normalised by the disorder strength. **c**, Multifractality of the eigenfunctions. The τ_q exponent has been determined from a power-law fit of the generalised inverse participation ratio Eq. (S2), averaged over all the grey states, as a function of N. For the fitting procedure, several values of N from 50 to 2000 have been considered. The dashed line is the analytical result obtained for the TC Hamiltonian with random uniformly distributed atomic energies in the thermodynamic limit [1]. In all panels, we neglect the polaritons, and we normalised the grey-state eigenfunctions to their total probability on the atoms.

In Fig. S1**a**, the squared amplitudes of some representative eigenfunctions $|E\rangle$ on the atoms $|i\rangle = \hat{\sigma}_i^+ |G\rangle$ are shown as a function of the atom index i. Different disorder strengths have been considered (see legend in panel **c**), which are comparable to the range covered by the experiments (see Fig. 2 in the main text). As one can see, the eigenfunctions are characterised by few highly occupied atoms, and many atoms with a small occupation probability, equally distributed in space. On the other hand, the occupation probabilities as a function of the atomic energies have a power-law dependence, as shown in Fig. S1**b**. These same features characterise the multifractal eigenstates of the TC Hamiltonian with random, uniformly distributed atomic energies, as shown in Ref. [1]. Then, following Ref. [1], we quantify the multifractal behaviour of the eigenfunctions by analyzing the scaling of their generalised inverse participation ratio with the system size,

$$\mathrm{IPR}_q(|\psi\rangle) = \sum_{i=1}^{N} |\langle i|\psi\rangle|^{2q} \propto N^{-\tau_q} \ . \tag{S2}$$

The definition in Eq. (S2) is valid for any normalised wavefunction $|\psi\rangle$, and here we consider the eigenfunctions $|\psi\rangle = |E\rangle$. The τ_q exponent is related to the multifractal dimension D_q by the relation [6]

$$D_q = d\frac{\tau_q}{q-1} \ , \tag{S3}$$

where d is the physical dimension of the system. In Fig. S1**c** the dependence of τ_q on the power q is shown for different disorder strengths. The numerical results match very well the analytical results of Ref. [1] (dashed line) up to some deviations around $q \approx 1/2$, due to the finite-size N. These results confirm that the eigenfunctions are multifractal with the correlated potential in Eq. (S1) that has been realised in the experiment described in the main text.

1.2 Modelling via Lindblad equation

Here, we provide further details on the model used to derive the relation between the atomic susceptibility χ_a and the population of the auxiliary state $P_\mathrm{A}(t)$, given in Eq. (8) of the Methods.

We model the experimental sequence of Secs. 7.2–7.4 via a Lindblad master equation, which allows us to derive an equation of motion for $P_\mathrm{A}(t)$ in terms of $\chi_\mathrm{a}(\Delta_\mathrm{pa})$. We work in a rotating frame generated by $N\Delta_\mathrm{pa}\hat{S}^z$, such that the Hamiltonian of Eq. (11) is time-independent, i.e., $\hat{\mathcal{V}}(t) \to \hat{\mathcal{V}} = \hat{\mathcal{V}}(0)$. The Lindblad equation is then given by

$$\partial_t\hat{\rho}(t) = -i\Big[\hat{H}_\mathrm{LMG} - N\Delta_\mathrm{pa}\hat{S}^z + \hat{\mathcal{V}}, \hat{\rho}(t)\Big] + \big(\mathcal{D}[\Gamma_\mathrm{g};\ \{\hat{\sigma}_i^-\}] + \mathcal{D}[\Gamma_\mathrm{a};\ \{|a\rangle\langle e|_i\}]\big)\hat{\rho}(t), \tag{S4}$$

where the superoperators $\mathcal{D}[\gamma'; \{\hat{L}_i\}]\hat{\rho}(t) \equiv \gamma' \sum_{i=1}^{N} \left(\hat{L}_i \hat{\rho}(t) \hat{L}_i^\dagger - \left\{\hat{L}_i^\dagger \hat{L}_i, \hat{\rho}(t)\right\}/2\right)$ describe dissipation at a rate γ', due to jump processes generated by $\{\hat{L}_i\}$. Specifically, the superoperators with rates Γ_g and Γ_a describe spontaneous decay of atoms from $|e\rangle$ to $|g\rangle$ and $|a\rangle$, respectively. The decay rates $\Gamma_\mathrm{g}, \Gamma_\mathrm{a}$ are branching ratios of the natural linewidth $\Gamma = 5.8 \times 2\pi\,\mathrm{MHz}$ of the D2 line of ^{6}Li, i.e., $\Gamma_\mathrm{g} + \Gamma_\mathrm{a} = \Gamma$. Spontaneous emission from $|a\rangle$ to $|g\rangle$ can be neglected on the timescales of the experiment, as for ^{6}Li it is forbidden by selection rules.

The presence of a single atom in state $|a\rangle$ drastically suppresses the transmission signal, due to the cavity's high cooperativity. The experiment must therefore be executed in a regime where at most one atom is in state $|a\rangle$ so as to avoid saturation of the transmission signal (see Fig. E2**c**). We therefore project the dynamics of Eq. (S4) onto the Hilbert subspace with at most one excitation and at most one atom in the auxiliary state $|a\rangle$. We then utilise a separation of scales to derive the equation of motion for $P_\mathrm{A}(t) \equiv \sum_{i=1}^{N} \langle a_i|\hat{\rho}(t)|a_i\rangle$ (where $|a_i\rangle \equiv |a\rangle\langle g|_i\,|G\rangle$): Within the time domain $t \gg (\Gamma/2)^{-1}$, all coherences as well as the SEM populations can be adiabatically eliminated from the rate equations of the remaining populations $p_G(t)$ and $\{\langle a_i|\hat{\rho}(t)|a_i\rangle\}_{i=1}^{N}$. Doing so, one finds that $\partial_t p_G(t) = -\partial_t P_\mathrm{A}(t)$ (conservation of atomic population), and to lowest order in $(\Gamma/2)^{-1}$

$$\begin{aligned} \partial_t p_G(t) &= -\Gamma_\mathrm{a} \sum_{m \in \mathrm{SEM}} \frac{|\mathcal{V}_{mG}|^2}{(\Gamma/2)^2 + (E_{mG} - \Delta_\mathrm{pa})^2} p_G(t) \\ &= -\frac{\Gamma_\mathrm{a}}{(\Gamma/2)^2} \left|\frac{g\Omega_\mathrm{p}}{\Delta_\mathrm{ca}}\right|^2 \chi_\mathrm{a}(\Delta_\mathrm{pa}) p_G(t). \end{aligned} \tag{S5}$$

To obtain the relation to $\chi_\mathrm{a}(\Delta_\mathrm{pa})$, as defined by Eq. (15), we have used that (within the rotating frame of $N\Delta_\mathrm{pa}\hat{S}^z$) the matrix elements $\mathcal{V}_{mG}$ follow from Eq. (12) as $\mathcal{V}_{mG} = \frac{g\sqrt{N}\Omega_\mathrm{p}}{\Delta_\mathrm{ca}} \langle m|\hat{S}^+|G\rangle$, and we have identified the Lorentzian response of Eq. (14), with linewidth $\gamma = \Gamma/2$.

For the initial conditions $p_G(0) = 1, P_\mathrm{A}(0) = 0$, we finally obtain the relation stated in Eq. (8),

$$P_\mathrm{A}(t_\mathrm{meas}) = 1 - \exp\left(-\frac{\Gamma_\mathrm{a}}{(\Gamma/2)^2} \left|\frac{g\Omega_\mathrm{p}}{\Delta_\mathrm{ca}}\right|^2 \chi_\mathrm{a}(\Delta_\mathrm{pa}) t_\mathrm{meas}\right). \tag{S6}$$

For further discussions of the properties of this relation, we refer the reader to Sec. 7.6 of the Methods.

1.3 Finite-size scaling of the minimal ferromagnetic gap in the large-detuning regime

Here, we analyse the finite-size scaling of the minimal ferromagnetic gap Δ_FM of the disordered LMG model in Eq. (2). For this, we first Kac normalise the

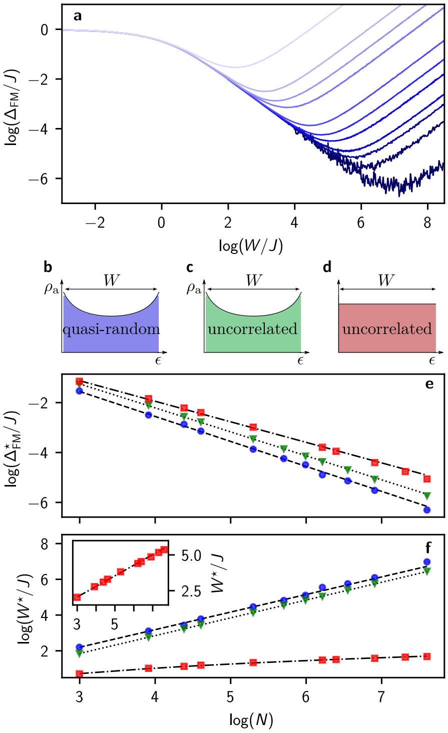

SUPPLEMENTARY FIG. S2 Finite-size scaling of the minimal ferromagnetic gap of the random LMG model. a, Disorder averaged ferromagnetic gap Δ_{FM}/J of the Kac-normalised random LMG Hamiltonian, as a function of disorder strength W/J. Only data for the quasi-disordered case is shown as a representative. Lighter to darker shades of blue correspond to increasing N from 20 to 2000. For each N, the minimal gap $\Delta^\star_{FM}/J$ and its location $W^\star/J$ are determined from a parabolic fit to the corresponding curve. **b**–**d**, Schematic distributions of the considered disorders: quasi-random and i.i.d. ϵ_i from ρ_a, and i.i.d. ϵ_i from uniform distribution, left to right. **e** and **f**, $\log\big(\Delta^\star_{FM}/J\big)$ and $\log(W^\star/J)$ versus $\log(N)$, respectively, for the quasi-random ρ_a (blue circles), uncorrelated ρ_a (green triangles), and uniform (red squares) disorder distributions. The errors in the parabolic fits are smaller than the size of the markers. From linear fits to the data points for the quasi-random (dashed) and the uncorrelated (dotted) ρ_a, we find $W^\star/J \propto N^\alpha$ with $\alpha \approx 0.98$ and 0.99, respectively, indicating linear dependence. For the uniform disorder distribution, we fit a logarithmic curve (dot-dashed), which suggests that $W^\star/J \propto \log(N)$. This is verified by the linear fit (dot-dashed) in the inset of **e**, where the y-axis depicts $W^\star/J$ instead of $\log(W^\star/J)$. In contrast, the minimal ferromagnetic gap $\Delta^\star_{FM}/J$ scales as $N^{-\beta}$ with $\beta \approx 1.00$, 0.96, and 0.82 for the considered disorders, respectively.

all-to-all spin-exchange interaction term in the Hamiltonian by N^{-1}, which renders the model extensive. In contrast to the experimental scenario, Kac normalisation is necessary to theoretically analyse any critical behaviour stemming from the competition between different terms in the Hamiltonian. Under this rescaling, the zero-disorder gap is $\Delta_{\rm FM}/J = 1$, which decreases to a minimal value $\Delta^\star_{\rm FM}/J$ as the disorder strength W/J is increased. We denote the disorder strength at which this minimum is realised as $W^\star/J$. The minimal gap is indicative of significant changes in the ground-state properties in a finite-size

system. We perform a finite-size scaling of $\Delta^{\star}_{\mathrm{FM}}/J$ and $W^{\star}/J$ for the system with (i) quasi-random disorder [sampled from the correlated energy potential of Eq. (S1)], and compare it with (ii) uncorrelated disorder with distribution ρ_{a} (as defined in Sec. 2 of the main text), and (iii) uniform distributions (see Fig. S2).

The dependence of Δ_{FM}/J on W/J is shown in Fig. S2**a** for the correlated disorder, which (adapting for different scalings, see below) is representative also of the two other studied cases. The minimal gap $\Delta^{\star}_{FM}/J$, and its location $W^{\star}/J$, are determined by fitting a parabola to the minimum of the curves. For all three disorder distributions, the gap $\Delta^{\star}_{\mathrm{FM}}/J$ decreases as $N^{-\beta}$ (see Fig. S2**e**), indicating that the gap disappears in the thermodynamic limit. The gap location $W^{\star}/J$, however, scales linearly with system-size ($\propto N^{\alpha}$ with $\alpha \approx 1$) for the uncorrelated and correlated ρ_{a} (see Fig. S2**f**). In contrast, $W^{\star}/J \propto \log N$ for the uncorrelated uniform disorder (inset of Fig. S2**f**), which is consistent with the vanishing Richardson's superconducting gap, which was estimated for uniform disorder from the mean level spacing [7]. These findings are in agreement with the generic behaviour of the critical disorder strength W_{c} for the Anderson localisation transition in models with a high connectivity, which increases with the number of connections [8–11]. For example, in a d-dimensional hypercube with coordination number $z \sim 2^d$, one finds $W_c \propto d \log d$ [10]. By visualising the all-to-all connectivity realised in the large-detuning regime as a hypercube with dimension $d \sim N$, we can expect $W_{\mathrm{c}} \propto \log N$ to leading order. This similarity indicates the significant change in localisation properties that a finite system experiences around $W^{\star}/J$, which is also supported by the decreasing trend in the PR (similar to Fig. 4). Correlations in the disorder tend to delocalise the system more, consistent with our finding in Fig. S2**f**. In the thermodynamic limit, however, the infinitely-connected system does not support a localisation transition.

References

[1] Dubail, J., Botzung, T., Schachenmayer, J., Pupillo, G., Hagenmüller, D.: Large random arrowhead matrices: Multifractality, semilocalization, and protected transport in disordered quantum spins coupled to a cavity. Phys. Rev. A **105**(2), 023714 (2022). https://doi.org/10.1103/PhysRevA.105.023714

[2] Harper, P.G.: Single Band Motion of Conduction Electrons in a Uniform Magnetic Field. Proc. Phys. Soc. A **68**(10), 874–878 (1955). https://doi.org/10.1088/0370-1298/68/10/304

[3] Aubry, S., André, G.: Analyticity breaking and Anderson localization in incommensurate lattices. Ann. Israel Phys. Soc **3**(133), 18 (1980)

[4] Anderson, P.W.: Absence of Diffusion in Certain Random Lattices. Phys. Rev. **109**, 1492–1505 (1958). https://doi.org/10.1103/PhysRev.109.1492

[5] Deng, X., Ray, S., Sinha, S., Shlyapnikov, G.V., Santos, L.: One-Dimensional Quasicrystals with Power-Law Hopping. Phys. Rev. Lett. **123**, 025301 (2019). https://doi.org/10.1103/PhysRevLett.123.025301

[6] Evers, F., Mirlin, A.D.: Anderson transitions. Rev. Mod. Phys. **80**, 1355–1417 (2008). https://doi.org/10.1103/RevModPhys.80.1355

[7] Celardo, G.L., Kaiser, R., Borgonovi, F.: Shielding and localization in the presence of long-range hopping. Phys. Rev. B **94**, 144206 (2016). https://doi.org/10.1103/PhysRevB.94.144206

[8] Abou-Chacra, R., Thouless, D.J., Anderson, P.W.: A selfconsistent theory of localization. J. Phys. C: Solid State Phys. **6**(10), 1734–1752 (1973). https://doi.org/10.1088/0022-3719/6/10/009

[9] Mirlin, A.D., Fyodorov, Y.V.: Distribution of local densities of states, order parameter function, and critical behavior near the Anderson transition. Phys. Rev. Lett. **72**, 526–529 (1994). https://doi.org/10.1103/PhysRevLett.72.526

[10] Tarquini, E., Biroli, G., Tarzia, M.: Critical properties of the Anderson localization transition and the high-dimensional limit. Phys. Rev. B **95**, 094204 (2017). https://doi.org/10.1103/PhysRevB.95.094204

[11] Roy, S., Logan, D.E.: Localization on Certain Graphs with Strongly Correlated Disorder. Phys. Rev. Lett. **125**, 250402 (2020). https://doi.org/10.1103/PhysRevLett.125.250402
